# Friend or foe: classifying collaborative interactions using fNIRS

**DOI:** 10.3389/fnrgo.2023.1265105

**Published:** 2023-10-11

**Authors:** Lucas Hayne, Trevor Grant, Leanne Hirshfield, R. McKell Carter

**Affiliations:** ^1^Computer Science, University of Colorado, Boulder, CO, United States; ^2^Psychology and Neuroscience, University of Colorado, Boulder, CO, United States

**Keywords:** competition, cooperation, fNIRS, machine learning, classification, neuroimaging, wearable sensors, collaborative problem solving

## Abstract

To succeed, effective teams depend on both cooperative and competitive interactions between individual teammates. Depending on the context, cooperation and competition can amplify or neutralize a team's problem solving ability. Therefore, to assess successful collaborative problem solving, it is first crucial to distinguish competitive from cooperative interactions. We investigate the feasibility of using lightweight brain sensors to distinguish cooperative from competitive interactions in pairs of participants (N=84) playing a decision-making game involving uncertain outcomes. We measured brain activity using functional near-infrared spectroscopy (fNIRS) from social, motor, and executive areas during game play alone and in competition or cooperation with another participant. To distinguish competitive, cooperative, and alone conditions, we then trained support vector classifiers using combinations of features extracted from fNIRS data. We find that features from social areas of the brain outperform other features for discriminating competitive, cooperative, and alone conditions in cross-validation. Comparing the competitive and alone conditions, social features yield a 5% improvement over motor and executive features. Social features show promise as means of distinguishing competitive and cooperative environments in problem solving settings. Using fNIRS data provides a real-time measure of subjective experience in an ecologically valid environment. These results have the potential to inform intelligent team monitoring to provide better real-time feedback and improve team outcomes in naturalistic settings.

## 1. Introduction

### 1.1. Team competition and cooperation

Professionals across all disciplines compete and cooperate every day to achieve personal and collective goals in their work. These personal and collective goals are often achieved through teams. In teams, multiple individuals interact to complete tasks in an organizational setting (Kozlowski and Bell, 2013). Competition arises in teams when two or more individuals interact to achieve mutually exclusive goals. Whereas cooperation occurs when individuals interact to achieve common goals. As one of the oldest research topics in social psychology, a huge number of studies relate competition, cooperation, and individualistic effort to individual and team outcomes, often with contradictory findings. In a meta analysis of these studies, Johnson (2003) found that the average individual in a cooperative setting outperforms their peers working in either competitive environments or alone. In contrast, other studies have found that cooperative teams are less productive than groups of individual workers (Young et al., 1993).

Apart from achievement, cooperation and competition can have a significant effect on psychological outcomes experienced by team members. Psychological outcomes are an important assessment of not only an individual's health within a team, but also a strong predictor of team success. Successful teams are composed of confident individuals with positive interpersonal relationships (Tjosvold et al., 2003). While meta analysis reveals an overall positive effect of cooperation on interpersonal relationships, compared to competition (Johnson, 2003), other studies have found that competition can improve team satisfaction under certain settings (Tjosvold et al., 2003; Abraham et al., 2019).

In addition to the workplace, the classroom is another ideal setting to study team dynamics. In a large meta-analysis of North American schools, Johnson et al. (1981) found that cooperation between students led to significantly increased achievement compared to individualistic competition. Canning et al. (2020) show that competitive classroom environments have a tendency to increase the prevalence of imposter feelings in first generation college students and lead to negative education outcomes Ames and Archer (1988) show that when students focus on outperforming their peers, they develop more negative feelings regarding their ability in the face of failure. Urdan (2004) found that classroom environments that emphasized competition led to self-handicapping behaviors amongst students. In a review, Meece et al. (2006) describe that generally competition decreases motivation among students. Indeed, an overemphasis on competition in college classrooms could be a contributing factor to the lack of collaborative problem solving skills amongst college graduates (Fiore et al., 2018).

### 1.2. Behavioral measures of team cooperation and competition

Based on these diverse, and often contradictory behavioral findings, there is a clear need in the teams literature to better understand and measure competition and cooperation. Although competition and cooperation can have a diverse and considerable effect on team outcomes, measuring the degree to which individuals are engaging in these behaviors can be difficult. To measure competition, researchers often extract features from interactions between individuals (Abraham et al., 2019). This process can involve many hours of training research assistants to record and code team interactions. Coding frameworks themselves can be subject to bias in the case where coders disagree on how to categorize interactions. To alleviate these issues researchers often turn to surveys to gauge individual perceptions of competition and cooperation in teams (Canning et al., 2020). However, surveys sent during task time can distract individuals and derail interactions. Surveys sent after team interactions have the opposite problem in that they fail to capture accurate, real-time, and granular information regarding individual perceptions of specific interactions (Dang et al., 2020).

Adapted subjective measure systems, such as the Team Workload Questionnaire, have been proposed and adopted for teams that attempt to mitigate some of the issues detailed above (Sellers et al., 2014). But these surveys, similarly to their individual subjective measure counterparts, can only be elicited after a teaming task has been completed, limiting the ability of researchers to determine how, why, or when a teaming scenario may have been derailed. By instead focusing on the real time physiological activity within the brain where these types of states are elicited (Dehais et al., 2020), researchers have a better opportunity to collect a real time measure that is not as subject to some of the constraints of subjective measures.

### 1.3. Neurophysiological sensors for measuring cooperation and competition

One way to capture accurate, real-time, and granular information from interactions between individuals is through neuroimaging. Neuroimaging has been a useful tool for studying the neural and cognitive bases of competition and cooperation. In competition and cooperation, two major cognitive processes play a role: executive and social functioning. Executive function refers to the ability to plan future actions, suppressing some while actualizing others. Executive function, in the case of competition and cooperation, has been shown to primarily recruit the prefrontal cortex, or more specifically the superior frontal gyrus (Decety et al., 2004). Social processing, on the other hand, primarily involves predicting the future actions of others. These social functions are typically associated with the temporal parietal junction which has been shown to activate in both cooperative (Abe et al., 2019) and competitive (Decety et al., 2004) interactions. These foundational studies have employed fMRI machines which are not well suited to capturing neural data in a naturalistic setting.

For monitoring interactions in naturalistic settings, portable and economic sensors have been developed which can also alleviate the biases associated with measuring individual interactions based on surveys. Using these sensors, researchers are in the process of developing tools for monitoring other individual states like engagement and affect (Dich et al., 2018; Henderson et al., 2020; Sümer et al., 2021). These methods currently use widely available sensors like cameras, microphones, and galvanic skin response wristbands. However, brain imaging technology is advancing rapidly and could soon be accessible in workplaces and classrooms (Davidesco et al., 2021). Here, we use functional near infrared spectroscopy (fNIRS), a non-invasive sensor for measuring blood-flow in the cerebral cortex. The fNIRS device provides a portable alternative to fMRI, the gold standard for high spatial resolution brain imaging, for use in naturalistic settings. This makes fNIRS a promising candidate for measuring team collaborative experiences. Our goal is to assess the feasibility of fNIRS for detecting and distinguishing competitive and cooperative interactions. These results can be used to inform intelligent agents (Brawner and Goldberg, 2012) and improve workplace and classroom monitoring.

Although previous studies have investigated competition and cooperation using fNIRS, they tend to focus on synchrony, or coherence, between brains as users interact. This kind of between-brain analysis, called “hyperscanning”, was first introduced twenty years ago as a promising new method for studying social cognition in fMRI (Montague et al., 2002; Misaki et al., 2021). Early hyperscanning studies used fMRI to assess participant interactions, but later became a popular analysis tool for portable neuroimaging devices like EEG (Dikker et al., 2017) and fNIRS (Pinti et al., 2020) (see Tsoi et al., 2022 and Czeszumski et al., 2020 for a review of hyperscanning). In fact, one of the first studies to simultaneously record two individuals using fNIRS investigated cooperation and competition as participants played a computer game. In that study, Cui et al. (2012) determined that the coherence between individual signals in the superior frontal cortex increases in cooperation compared to competition. Coherence results in the frontal cortex during cooperation have also been found using EEG (Babiloni et al., 2006). In contrast, Liu et al. (2017) found increased synchrony in the inferior parietal lobule during competition compared to cooperation. These studies provide evidence that cooperative and competitive states can be distinguished using synchrony.

However, in this study we do not evaluate synchrony for three reasons. First, we had hoped to target more easily interpreted neural metrics. For instance, coherence has been found between participants presented with the same stimuli but who do not interact (Burgess, 2013). Separating the neural effects of interaction from those elicited by a shared stimulus requires complex experimental designs (Hamilton, 2021). Those experimental designs are not employed here. Instead, we are interested in evaluating interpretable neural features for distinguishing cooperative and competitive interactions. As such, we devote a section titled “Interpretability” to deciphering the features used in our machine learning analysis. The second reason for focusing on activation rather than synchrony is our aim to develop features for distinguishing individual experiences of cooperation and competition. Synchrony is more difficult to use as a feature for distinguishing individual experiences of cooperation and competition because it requires the simultaneous recording of more than one individual at a time. For evaluating a single individual at a time (e.g., a student interacts with an AI-based tutoring system, a pilot conducts a formation flight on a simulator) activity-based features are more feasible than their synchony-based counterparts. One previous fNIRS study has measured activity differences in competitive and cooperative motor movement tasks. Chen et al. (2020) showed increased activation for social brain regions in competition and cooperation with greater increases in competitive conditions. Finally, to clarify the function of these social brain regions, we chose to use an activity-based rather than synchrony-based analysis. With this activity-based analysis, we specifically target the temporal parietal junction (TPJ) which “contributes to decision making specifically when there is a social context relevant for current behavior” according to the nexus model of the TPJ (Carter and Huettel, 2013). Using features extracted from the TPJ and other relevant brain regions, we aim to investigate the usefulness of fNIRS for predicting individual experiences of competition and cooperation.

The task we chose for this study is the Balloon Analogue Risk Task (BART). Since its introduction twenty years ago, researchers have used the BART in the lab to measure real-world risk-taking behavior (Lejuez et al., 2002). A small number of studies have investigated the BART using fNIRS. Cazzell et al. (2012) published the first study to use fNIRS in conjunction with the BART and aim primarily to validate findings from fMRI studies and identify gender differences in win and loss conditions. In support of previous fMRI research, they find that increased bilateral dorsolateral prefrontal cortex (dlPFC) activity correlates with risk aversion and that females show more dlPFC activity during loss conditions. In a follow-up study, Li et al. (2017) performed a similar experiment on older adults and found decreased dlPFC activation in older adults during winning conditions. Using the BART, Huhn et al. (2019) find useful fNIRS features in the dlPFC for predicting cocaine relapse amongst drug recovery patients. In all of these BART studies, participants complete the task alone. However, here we use a competitive version of the BART (Fairley et al., 2019) and add our own cooperative condition.

### 1.4. Contributions and novelty

We measure brain activity during competition and cooperation using fNIRS as participants interact through the BART game. We find that both cooperation and competition increase activity in social areas of the brain. Although activity in these areas increases in both competitive and cooperative conditions, it increases much more in competitive conditions. Guided by these activation differences, we train support vector classifiers based on features extracted from social, motor, and executive regions of the brain. We show that social region features significantly outperform other feature sets for discriminating competitive, cooperative, and alone conditions.

We present the following contributions:

- We characterize brain activation in competitive and cooperative scenarios using unobtrusive functional near-infrared spectroscopy sensors in an ecologically valid decision-making game involving uncertain outcomes.
- We demonstrate that fNIRS features from social regions of the brain outperform other features for distinguishing collaborative interactions, which paves the way for the development of more reliable and unobtrusive tools for workplace and classroom monitoring of team interactions.
- We perform additional analysis of the features salient to competitive and cooperative conditions to find that both cooperative and competitive conditions increase activity in the social regions of the brain but that competition shows larger increases in social areas than cooperation.

## 2. Methods

The following sections outline our data collection procedure, dependent measures, machine learning paradigm, and interpretability procedure. We fit machine learning classifiers to features extracted from our sensors and evaluated the usefulness of those features for distinguishing collaborative interactions. Additionally, to better interpret our machine learning results, we performed a univariate analysis which revealed how fNIRS activity levels changed during competition and cooperation.

### 2.1. Data collection

#### 2.1.1. Participants

One hundred and sixteen participants enrolled in our study. Email screenings excluded participants under eighteen and those with a history of seizures. Dyads of participants who had met previously were excluded from the study, or were reassigned to different partners. Written informed consent was obtained from all subjects in a protocol approved by a local university institutional review board. During analysis, thirty two participants were excluded based on fNIRS data quality issues, leaving eighty four healthy volunteers (age 31 ± 13.8 years, 44 females, 39 males, 1 unanswered) in the final analysis.

#### 2.1.2. Task and conditions

**Figure 1:**
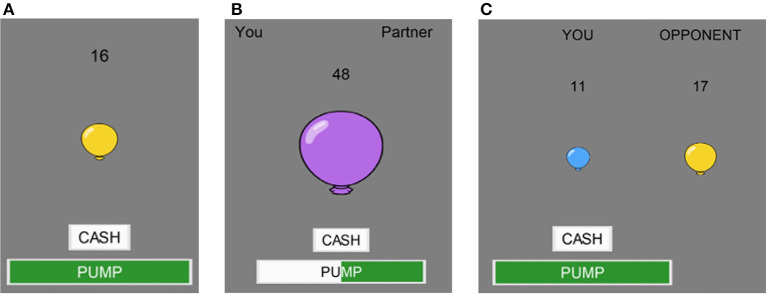
Gameplay. Examples from the **(A)** solo, **(B)** cooperative, and **(C)** competitive BART.

Participants played a version of the BART alone, which we call solo BART (Figure 1A), a cooperative BART, and a competitive BART. For the entire experimental session, Dyads completed one block of the solo BART at the beginning and at the end of the session. In the middle of the session, dyads completed two cooperative BART blocks and two competitive BART blocks in a random order, for a total of 6 blocks (one solo block, two competitive blocks, two cooperative blocks, followed by one final solo block). Each block, regardless of condition, lasted approximately seven minutes and thirty seconds (participants would finish their final balloon once the timer had been met, so block times would vary slightly). Rest conditions, which had the participant stare at a fixation cross for a period of 30 seconds, immediately preceded each block.

For all BART conditions, Participants complete the BART alone by pumping a virtual balloon on a computer screen. The balloon randomly pops after a predetermined, but unknown to the participant, number of pumps. Participants accrue tokens for each pump and can choose when to cash in their tokens any time before the balloon pops. If the balloon pops, participants lose all their accumulated tokens. During the solo BART condition, participants play alone, where they are able to pump the balloon and cash out tokens without any input from the other user (Figure 1A)

In the cooperative BART condition (Figure 1B), both participants must agree on an action in order for that action to occur. Each participants' action is registered on the screen for both players to see. If participants cannot agree on an action after ten seconds, then the balloon automatically cashes in. If the shared balloon pops, then both participants earn zero tokens. When a balloon is cashed in, participants split the total number of accumulated tokens.

In the competitive BART condition (Figure 1C), each participant sees both their own balloon and their opponents balloon on the screen. Each participant is free to pump and cash their own balloon whenever they like and can see the pumping and cashing behavior of their opponent. If both participants balloons pop, then neither person earns any tokens. If both participants cash in, then the person who cashed in with more tokens earns all their accumulated tokens and their opponent earns nothing. In the case of a tie, participants split the earnings.

#### 2.1.3. Experiment procedure

The experiment was conducted with a dyad and two research assistants. Upon arrival, participants signed consent forms, were guided to separate data acquisition rooms, and and through the use of a self-paced guided tutorial learned about the experimental conditions while the tutorial provided real time feedback to participants. Upon completion of the tutorial, researchers configured the fNIRS devices for each participant. Cap alignment was verified based on the 10-20 location of Fpz and data quality was checked. All experiment materials were presented using PsychoPy.

### 2.2. Dependent measures

#### 2.2.1. Behavioral data

For each participant and condition the adjusted number of pumps, or the average number of pumps during cashed out trials was calculated from keypresses collected during gameplay.

#### 2.2.2. fNIRS

fNIRS data was collected in both participants using two continuous-wave NIRSport2 devices (NIRx Medical Technologies, LLC) implemented wirelessly, allowing for brain measurement in operational settings. The devices emitted light from LED sources at wavelengths of 760 nm and 850 nm corresponding to oxygenated hemoglobin and deoxygenated hemoglobin concentrations, respectively. We collected the data at a sampling rate of 10 Hz using Aurora fNIRS, the NIRx data acquisition software. The fNIRS cap contained 15 sources, 16 detectors, and 42 channels covering bilateral frontal cortex, motor cortex, and the temporal parietal junction (TPJ).

**Figure 2:**
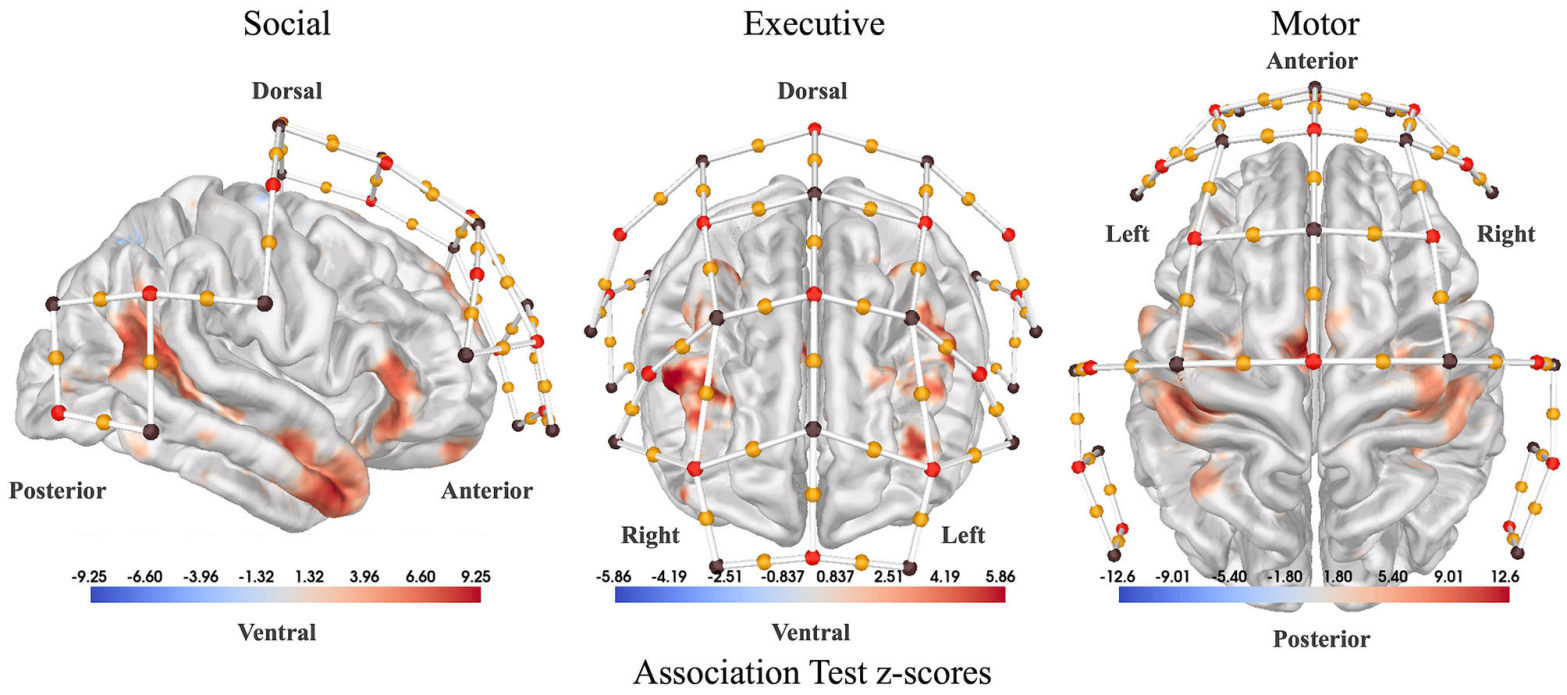
Sensor placement. Association test statistical maps from *NeuroSynth.org* used as a guide to place our fNIRS channels (yellow dots). **(Left)** Association test z-scores between the term “social” used in abstracts compiled by *NeuroSynth.org* and voxel activations reported by the corresponding paper (right lateral view). Association test z-scores are generated from a two-way ANOVA aimed at detecting a non-zero association for a particular voxel and term pair. The association test measures how much more likely each region is to be active in cases where the term of interest appears in the abstract versus when it does not appear. So, in the case of “social,” the red regions activate more consistently in studies with the term “social” in the abstract. **(Center)** Association test z-scores for the term “executive” (anterior view). **(Right)** Association test z-scores for the term “motor” (dorsal view). Red dots denote fNIRS sources, brown dots denote fNIRS detectors, and yellow dots represent channels in between each source-detector pair.

The locations of fNIRS channels on the scalp were determined using custom software to assess the relevance of specific regions of the cortex for our study. To generate the channel locations, first association test maps were downloaded from *Neurosynth.org* for three relevant search terms: “social”, “executive”, and “finger tapping”. Using these maps, weighted centroids were calculated on the cortex to mark the most relevant areas. Then, the closest fNIRS channels in the 10-20 coordinate system to each centroid were added to the headcap. Figure 2 shows fNIRS channel locations overlaid on NeuroSynth association maps. During the classification of participant interactions, features were extracted from channels over each of the three regions: “social”, “executive”, and “finger tapping”.

### 2.3. Analysis

#### 2.3.1. Behavioral analysis

To assess the effectiveness of each experimental manipulation, we analyze adjusted pumps, or the average number of pumps for all cashed out balloons. Two-sided T-tests were performed between the distributions of average adjusted pumps in each condition. We hypothesize that participants pump significantly less in the competitive condition compared to the solo BART.

### 2.4. General linear model

We fit a general linear model (GLM) following the procedure outlined in Meidenbauer et al. (2021). fNIRS data was processed using the *NIRS Brain AnalyzIR Toolbox* (Santosa et al., 2018). Raw light intensity data was downsampled to 4 Hz, converted into optical density, then converted into oxygenated (HbO) and deoxygenated (HbR) hemoglobin concentrations via the modified Beer-Lambert law (Strangman et al., 2003). After the conversion to hemoglobin, subject-level statistics were calculated. The autoregressive iteratively weighted least squares algorithm (AR-IRLS) was run in conjunction with the general linear model (GLM) to alleviate the statistical difficulties associated with the fNIRS signal. The AR-IRLS algorithm prewhitens the signal to decorrelate the noise and iteratively downweights outliers associated with motion artifacts in the data (Barker et al., 2013) (see Huppert, 2016 for more details). We selected a canonical HRF basis set for this analysis.

### 2.5. Machine learning

#### 2.5.1. Brain activity features

Brain activity features were computed by coding each block as a different stimuli and fitting a subject-level GLM to produce coefficients for each participant, channel, and block combination (see Section 2.4 for details on subject-level GLM). Channels located over the “social” region according to Figure 2 Left were grouped together. Similarly, channels located over the “executive” and “motor” regions according to Figure 2 Right, Figure 2 Center respectively were grouped together (see Appendix for details). These regions produced three feature sets which were evaluated independently for their ability to distinguish competitive, cooperative, and solo gameplay.

#### 2.5.2. Models

Support vector machines (SVMs) were used to assess the performance of each feature set for distinguishing cooperative and competitive interactions. We performed nested cross validation across participants such that each participant was placed in either the training or test set, but not both. At the beginning of each outer loop of nested cross validation, 20% of the data was reserved for a test set. Traditional 5-fold cross validation was then performed on the remaining 80% of the data (inner loop) to optimize the hyperparameters (L2 weight penalty, kernel choice). The best performing model in the inner loop was then refit on all 80% of the data and evaluated on the held out test set. The outer loop was repeated 50 times to evaluate 50 models on 50 randomly chosen test sets from the data (see Varoquaux et al., 2017 for more details on nested cross validation). Nested cross validation was performed separately for each feature set (“social”, “motor”, and “executive”) to distinguish competitive vs. solo, cooperative vs. solo, and competitive vs. cooperative. T-tests conducted between model accuracies determined whether one feature set significantly outperformed another on a given classification task. All brain activity features were standardized based on the training set. We used *scikit-learn* for all our machine learning models.

#### 2.5.3. Interpretability

After the subject-level analysis (Section 2.4), group-level statistics were calculated. Group-level statistics were calculated using a mixed effects model to determine the effect of each condition controlling for subject. The group-level statistical analysis uses the full covariance from the subject-level analysis to perform weighted least squares regression. The results of the group-level analysis were used for group contrasts between conditions at each channel. We use statistical maps to report group-level contrast activations based on Benjamini-Hochberg *p*-values (Benjamini and Hochberg, 1995).

## 3. Results

### 3.1. Behavioral analysis

Participants pump significantly less in the competitive condition (t=6.34, p < 0.001) and significantly more in the cooperative condition (t=2.41, p < 0.05) compared to the solo BART.

### 3.2. Machine learning models using fNIRS data

SVM models based on features extracted from each participant's fNIRS data in a nested cross-validation procedure (inner 5 fold split, outer 50 random tests) perform above chance (>50%): cooperative vs. competitive 55.3%; cooperative vs. solo 56.5%; competitive vs. solo 60.2%. Although lower than we hoped, these accuracies are comparable to other fNIRS studies which predict complex mental states (Gateau et al., 2015).

**Figure 3:**
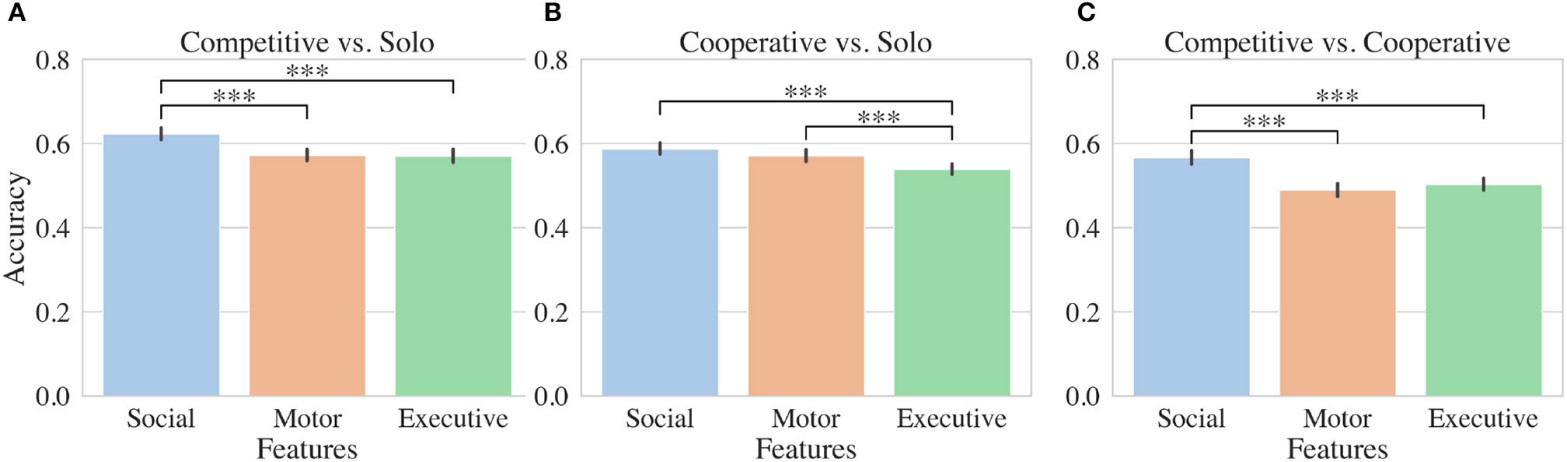
Machine learning results. Accuracy results for SVMs evaluated using either social, motor, or executive fNIRS features. Binary classifiers were trained to distinguish either **(A)** competitive and solo, **(B)** cooperative and solo, or **(C)** competitive and cooperative conditions. Stars indicate significant differences between accuracies for groups of classifiers trained in nested cross validation (see Section (2.5.2)).

To determine how executive, motor, and social features were contributing to the model, we constructed SVMs using a similar procedure but including each feature set independently. Features extracted from fNIRS channels positioned over social regions significantly outperformed executive and motor features for distinguishing competitive from solo conditions as shown in Figure 3A. SVMs trained on social features performed on average 5% better than classifiers trained on other feature sets. For distinguishing cooperative from solo conditions, both social and motor features outperformed executive features by 5% and 3% respectively (Figure 3B). In the case of distinguishing competitive and cooperative conditions, social features alone outperformed both motor and executive features by 7% and 6% respectively (Figure 3B).

### 3.3. Interpretation of the fNIRS model

**Figure 4:**
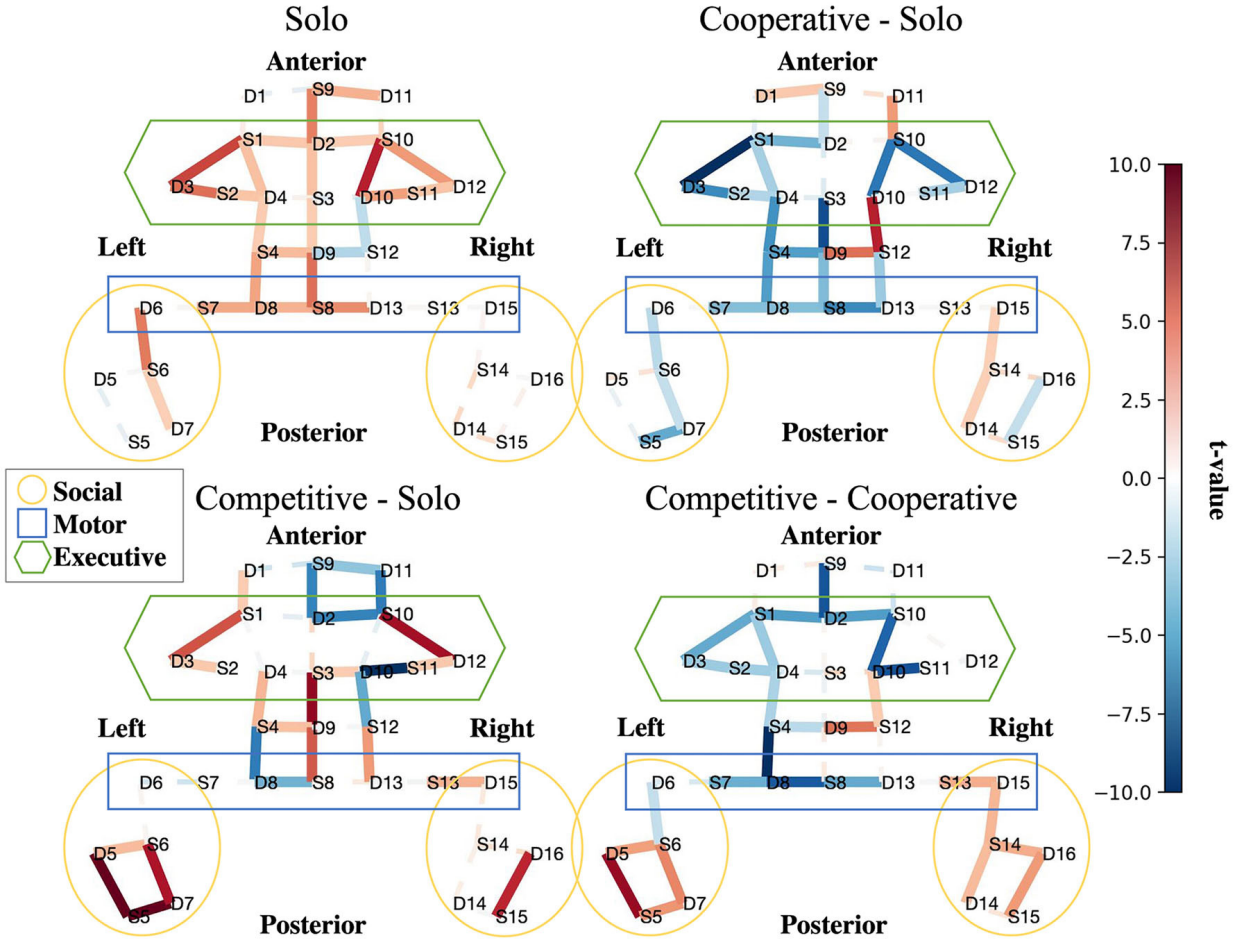
Interpretability. Social features are more active in competitive than cooperative and alone conditions. Contrast maps for solo BART vs. rest **(upper left)**, cooperative BART vs. solo BART **(upper right)**, competitive BART vs. solo BART **(bottom left)**, and competitive BART vs. cooperative BART **(bottom right)**. Maps are colored according to the direction and group-level reliability of the difference (*t*-values) in oxygenated hemoglobin concentrations (HbO) group-level HbO contrasts, red is more active (see Section (2.5.3)). Solid (dashed) lines are significant (insignificant).

The results of our group-level fNIRS analysis indicates whether particular features showed more activity or less activity in each social condition. We focus on four statistical contrast maps for interpretation: Solo BART vs. Rest (Figure 4, top left), Cooperative BART vs. Solo BART (Figure 4, top right), Competitive BART vs. Solo BART (Figure 4, bottom left), and Competitive BART vs. Cooperative BART (Figure 4, bottom right).

The first contrast (Figure 4, top left) reveals increased HbO activation in executive (bilateral dorsal lateral prefrontal cortex, dlPFC) and motor regions (primary motor cortex, pmc) during the solo BART condition compared to rest. Compared to the solo condition, the cooperative BART condition produced decreased HbO activation in the executive regions (bilateral dlPFC), motor regions, and social regions (left temporal parietal junction, TPJ) (Figure 4, top right).

The competitive BART condition produced decreased HbO activation in executive (bilateral dlPFC) and motor regions, but increased activation in social regions (bilateral TPJ) (Figure 4, bottom left). This difference is also evident in the final contrast between competitive and cooperative conditions, where the competitive BART condition produced significantly increased HbO activation in social regions (bilateral TPJ) compared to the cooperative condition.

## 4. Discussion

We present results investigating the feasibility of distinguishing collaborative interactions using fNIRS, a lightweight and portable brain sensor. Our aim was to identify brain activity features that distinguish cooperation and competition. We recruited a large sample of participants (N=84) to play an ecologically valid decision-making game with competitive, cooperative, and alone conditions. During these interactions, we used fNIRS sensors to measure executive, motor, and social regions based on a large meta analysis of neuroscientific studies. We found that support vector classifiers trained on these features could be used to distinguish social conditions. When comparing models constructed using subsets of features, social regions of the cortex significantly outperformed motor and executive features in distinguishing competitive, cooperative, and alone conditions. fNIRS features collected over social regions of the brain show promise as a means of distinguishing competitive and cooperative interactions in a problem solving settings.

When interpreting these models, we found that the alone task increased activation in motor and control, but not social regions of the brain. These increases reflect the use of cognitive functions implicated in risky decision making and are consistent with previous findings from both fMRI (Rao et al., 2008) and fNIRS (Cazzell et al., 2012; Li et al., 2017). Although social features were identified as bringing the largest improvement to our models, the interpretation analysis also revealed strong activity increases in all regions of the cortex during competitive interactions. We interpret this result as indicating that the competitive game was, on the whole, more taxing than the alone or cooperative game. The fact that interpretation analysis also showed increases in social regions indicates the competitive game is not just a more difficult task but a task that engages social processing. The overall increase in activity for the competitive condition echos findings from others showing that competition is more engaging than cooperation (Bitsch et al., 2018). This question is extremely important for the use of neural features of social engagement in models of team behavior. If problem solving is best in cooperative conditions, those may be best targeted by light but not strong activation in social areas of the brain.

The question still remains how these activation changes relate to hyperscanning results in cooperation in competition. Previous hyperscanning studies found increased coherence in the frontal cortex between participants engaged in cooperation (Babiloni et al., 2006; Cui et al., 2012). This frontal cortex coherence could be due to the fact that participants recruit the same executive processing resources when working on the same task together (Burgess, 2013). When working together, each person's actions become more predictable and the overall activity and coherence between social processing regions of the brain decreases. The opposite is true for competition where the overall activity and coherence in social processing regions increases (Liu et al., 2017). This increase occurs because both people become engaged in the difficult task of predicting their opponent's actions. The interplay between individual and hyperscanning brain dynamics should be investigated in future studies with games that simultaneously involve intra-team cooperation and inter-team competition. Until then, this study represents an important step forward in the field of neuroergonomics for collaborative teams.

We found that lightweight portable brain sensors show promise as tools for indexing collaborative experiences in teams. These tools can support usability testing on organizational materials designed for the workplace and the classroom. For instance, in recent years, there has been an increasing interest in the gamification of educational materials (González and Area, 2013). Gamified education materials inevitably provoke competition and cooperation amongst students with design elements like scores, leaderboards, teams, and missions. These game elements have varying effects on student satisfaction (Agapito et al., 2018; García Iruela et al., 2019). Similar incentives have been introduced in Fortune 500 companies to induce interteam competition (Young et al., 1993). How might these game elements affect individual experiences of competition and cooperation? As evidenced in this study, lightweight portable brain sensors can be used to evaluate individual experiences of gamified organizational materials in naturalistic team settings.

## 5. Limitations

Our study has several limitations which should be addressed in future work. First, our cooperative game may be less engaging than the competitive game. Although there is some evidence to support increased engagement for competition over cooperation in general, creating a more engaging cooperative game would provide interesting insights. For instance, intra-team cooperative games show promising results (Morschheuser et al., 2019). Second, we chose to capture only one social area, the TPJ. As our results indicate, future studies would benefit from recording more social areas shown in Figure 2. Apart from collecting data from more social regions, we believe that more participant data, shorter trials, different features, and better data cleaning would improve classification accuracies in future studies. We acknowledge the importance of synchrony-based features for classifying team interactions. Future studies would benefit from interpreting potentially useful synchrony-based features and reconciling those interpretations with those presented here. In addition, short-channel regressors should be used in future studies to further reduce sources of physiological noise following current best practices in the field (Tachtsidis and Scholkmann, 2016; Yücel et al., 2021). Third, although games, like the one used in this study, can be popular learning tools like those used in classroom teams (González and Area, 2013), future work should consider tasks that more directly reflect other common real-world team environments. Finally, a comparison of neuroimaging results with subjective measures of teammate perceptions is warranted. For example, do TPJ activation increases correlate with subjective ratings of task engagement or teammate competence? While unaddressed in the present work, these limitations point in exciting future directions for the study of teams using fNIRS.

## 6. Conclusion

Competitive and cooperative interactions in the workplace and the classroom can have a strong effect on team and individual outcomes. Measuring these interactions in real time has the potential to help organizations evaluate different goal structures and environments on individuals in teams. fNIRS provides a promising non-invasive tool for assessing individual interactions in teams in real time. This study identifies promising fNIRS features that can be used to distinguish competition and cooperation. These fNIRS features have exciting implications for teaming systems designed to improve team and individual outcomes.

## Data availability statement

The raw data supporting the conclusions of this article will be made available by the authors, without undue reservation.

## Ethics statement

The studies involving human participants were reviewed and approved by the University of Colorado Boulder Institutional Review Board. The participants provided their written informed consent to participate in this study.

## Author contributions

LH: Conceptualization, Data curation, Formal analysis, Investigation, Methodology, Software, Supervision, Validation, Visualization, Writing—original draft, Writing—review and editing. TG: Conceptualization, Data curation, Investigation, Methodology, Software, Supervision, Writing—review and editing. LH: Conceptualization, Funding acquisition, Methodology, Project administration, Resources, Supervision, Writing—review and editing. RC: Conceptualization, Funding acquisition, Investigation, Methodology, Project administration, Resources, Supervision, Writing—review and editing.
