# Supplementary material for: Friend or foe: classifying collaborative interactions using fNIRS
Source: Front Neuroergon. 2023 Oct 11;4:1265105. doi: 10.3389/fnrgo.2023.1265105 (PMC10790908; doi:10.3389/fnrgo.2023.1265105)
Supplement: Supplementary file 1 [file Table_1.pdf]

Table 1. Which source detector pairs (channels) were assigned to the three feature sets: "Social," "Executive," and "Motor."

Source	Detector	Feature Set
1	2	Executive
1	3	Executive
1	4	Executive
2	3	Executive
2	4	Executive
3	2	Executive
3	4	Executive
3	10	Executive
5	5	Social
5	7	Social
6	5	Social
6	7	Social
7	6	Motor
7	8	Motor
8	8	Motor
8	13	Motor
10	2	Executive
10	10	Executive
10	12	Executive
11	10	Executive
11	12	Executive
13	13	Motor
13	15	Motor
14	14	Social
14	16	Social
15	14	Social
15	16	Social

7 APPENDIX

Table 1 shows which source detector pairs from the fNIRS device were assigned to which feature sets. Each source detector pair forms a channel of brain activity data. Channels were assigned to feature sets based on custom software which placed channels over regions identified by meta analyses available from *Neurosynth.org*.
